# A Unique Case of a High-Grade Neuroepithelial Tumor With EML4-ALK Fusion in a Five-Month-Old

**DOI:** 10.7759/cureus.8654

**Published:** 2020-06-16

**Authors:** Oliver D Mrowczynski, Russell Payne, Cunfeng Pu, Robert Greiner, Elias Rizk

**Affiliations:** 1 Neurosurgery, Penn State Milton S. Hershey Medical Center, Hershey, USA; 2 Neurosurgery, University of Texas Southwestern Medical Center, Dallas, USA; 3 Pathology, Penn State Health Milton S. Hershey Medical Center, Hershey, USA; 4 Hematology-Oncology, Penn State Milton S. Hershey Medical Center, Hershey, USA

**Keywords:** high grade, eml4, alk, fusion, case report

## Abstract

We present a unique and challenging case of a high-grade neuroepithelial tumor with echinoderm microtubule-associated protein-like 4-anaplastic lymphoma kinase (EML4-ALK) fusion in a five-month-old child. This tumor was difficult to classify, with glial and ependymal features, reinforcing the impact of a molecular-based diagnosis in correct classification and management. The patient had two tumor resections and underwent chemotherapy following the Head Start trial treatment regimen. The patient remains well, with no residual disease on MRI 15 months after initial resection. Further studies are needed to determine the frequency of EML4-ALK fusions in these types of tumors and to optimize therapeutic protocols for children and adults, alike, suffering from this disease.

## Introduction

Chromosomal aberrations and fusions are proven to cause the genesis of numerous tumor types [1]. One such fusion is the echinoderm microtubule-associated protein-like 4 (EML4)-anaplastic lymphoma kinase (ALK) fusion gene [2]. ALK fusions were first identified with ALK-nucleophosmin (NPM) in non-Hodgkin’s lymphoma [3]. The EML4-ALK fusion was first described when discovering the underlying pathogenesis of non-small cell lung cancer (NSCLC) [4]. In addition to lung cancer, the EML4-ALK fusion has been found in breast and colorectal cancer [5,6].

The tumor in this case report is a high-grade neuroepithelial tumor and difficult to classify, with glial, as well as ependymal features. With regards to gliomas, infantile high-grade gliomas have been described to be ALK driven and congenital glioblastomas have also been described with an EML4-ALK fusion [7,8]. ALK fusions including Kinectin 1 (KTN1)-ALK and Coiled-coil domain containing 88C (CCDC88A)-ALK have been shown in ependymoma [9]. Here, we present a five-month-old child with a difficult to classify high-grade neuroepithelial tumor with an EML4-ALK fusion, containing glial and ependymal features.

## Case presentation

**Figure 1:**
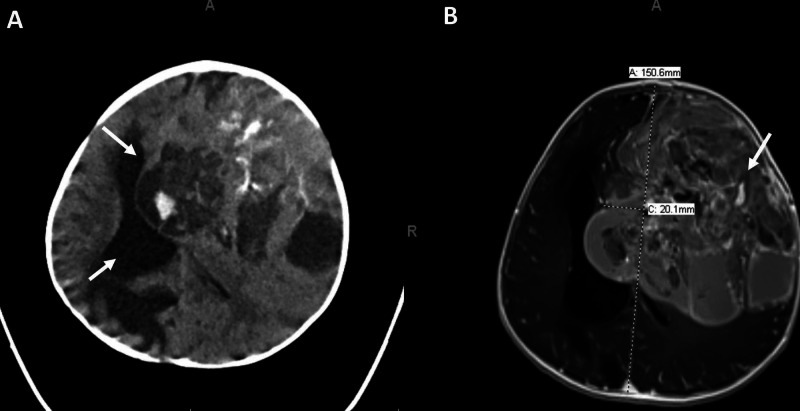
Diagnostic pre-resection imaging Diagnostic pre-resection imaging showing a frontotemporal heterogeneous and locally invasive mass causing obstructive hydrocephalous (arrow), 2 cm of midline shift (arrow), internal hemorrhage (arrow), and likely bony involvement of the left frontal bone. (A) Axial CT. (B) Axial MRI

**Figure 2:**
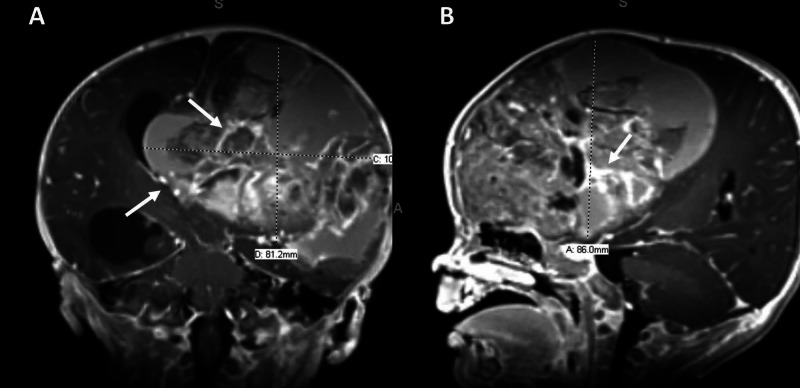
Diagnostic pre-resection imaging Diagnostic pre-resection imaging showing the same tumor in different vantage points again showing the obstructive hydrocephalous (arrow) and internal hemorrhage (arrow). (A) Coronal MRI. (B) Sagittal MRI

The patient is a five-month-old female with a history of full-term pregnancy without complications or significant medical history, who presented with increased fussiness and rapidly increasing head circumference. CT imaging demonstrated a frontotemporal mass with significant mass effect and hydrocephalus (Figure 1A). MRI demonstrated a left-sided, heterogeneously enhancing frontotemporal lesion with significant mass effect and intralesional hemorrhage (Figures 1B, 2A, 2B).

**Figure 3:**
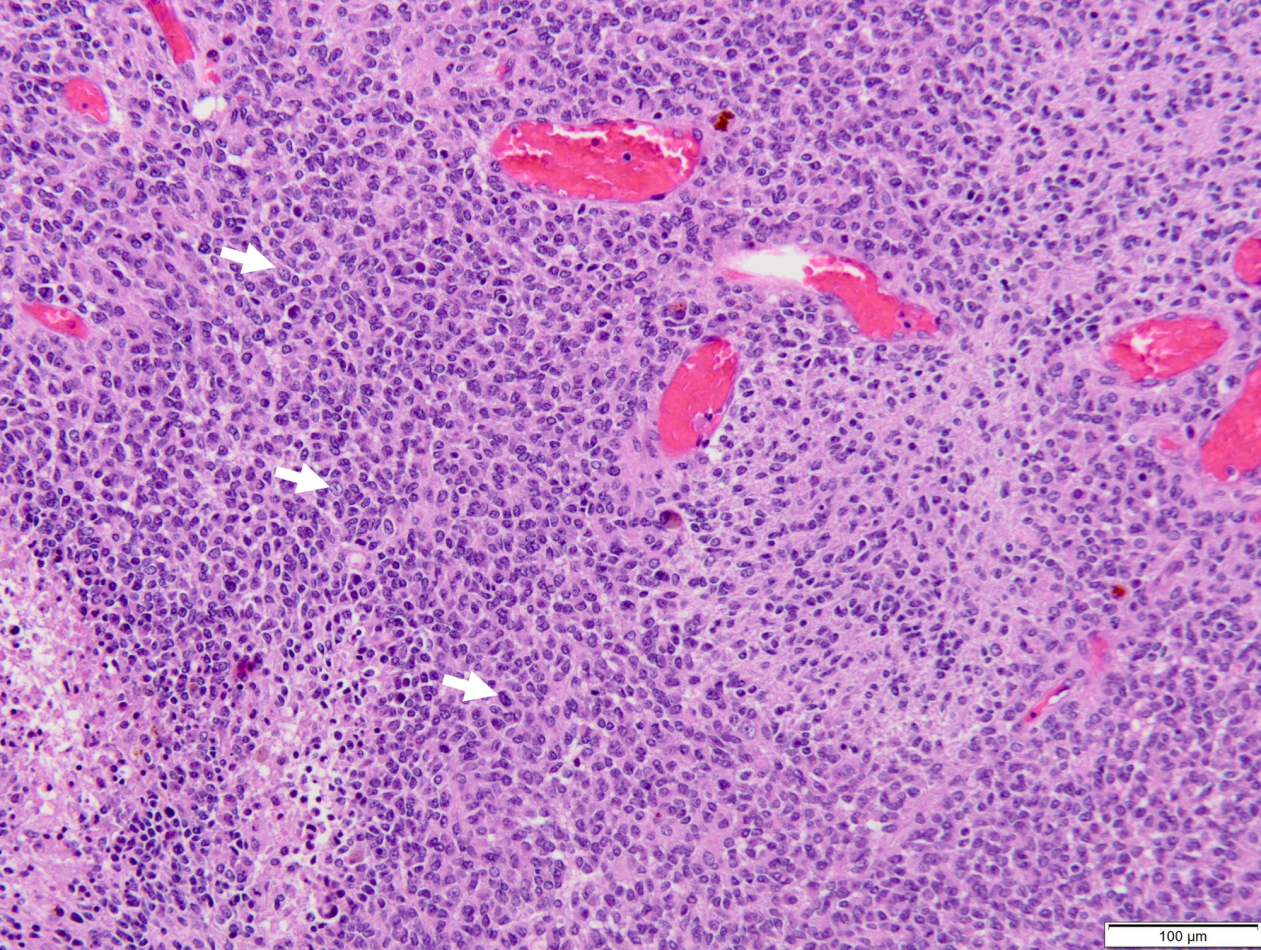
H/E stained section of the hypercellular tumor with necrosis The tumor cells show a high N/C ratio with frequent mitoses and apoptosis (arrow). By immunostain, some of the tumor cells show glial differentiation, while the tumor cells in the pale areas show evidence of neuronal differentiation. Abbreviations: H/E, hematoxylin and eosin; N/C, nuclear-cytoplasmic

She underwent a bifrontal craniotomy; however, only a partial resection was able to be obtained due to the vascularity of the mass and subsequent blood loss. Pathology demonstrated that the tumor cells showed a high nuclear-cytoplasmic (N/C) ratio with frequent mitoses and apoptoses. By immunostain, some of the tumor cells showed glial differentiation, while the tumor cells in the pale areas showed evidence of neuronal differentiation, which initially suggested an anaplastic ependymoma (Figure 3).

**Figure 4:**
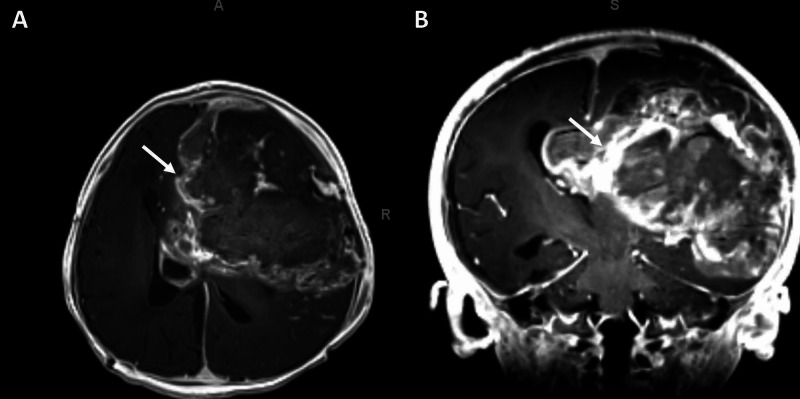
Post-resection imaging MRIs status post the first subtotal resection. (A) Axial MRI with residual peripheral areas of enhancement most predominantly in the left temporal lobe (arrow). (B) Coronal MRI showing intrinsic T1 hyperintense areas of hemorrhage within the surgical cavity (arrow)

However, upon further review and next-generation sequencing at Children's Hospital of Philadelphia, analysis deemed it to be a high-grade neuroepithelial tumor with EML4-ALK fusion and deletion of exons 14 to 19 of the ALK gene. Post-operative MRI showed areas of hemorrhage within the surgical cavity (Figure 4A, B).

**Figure 5:**
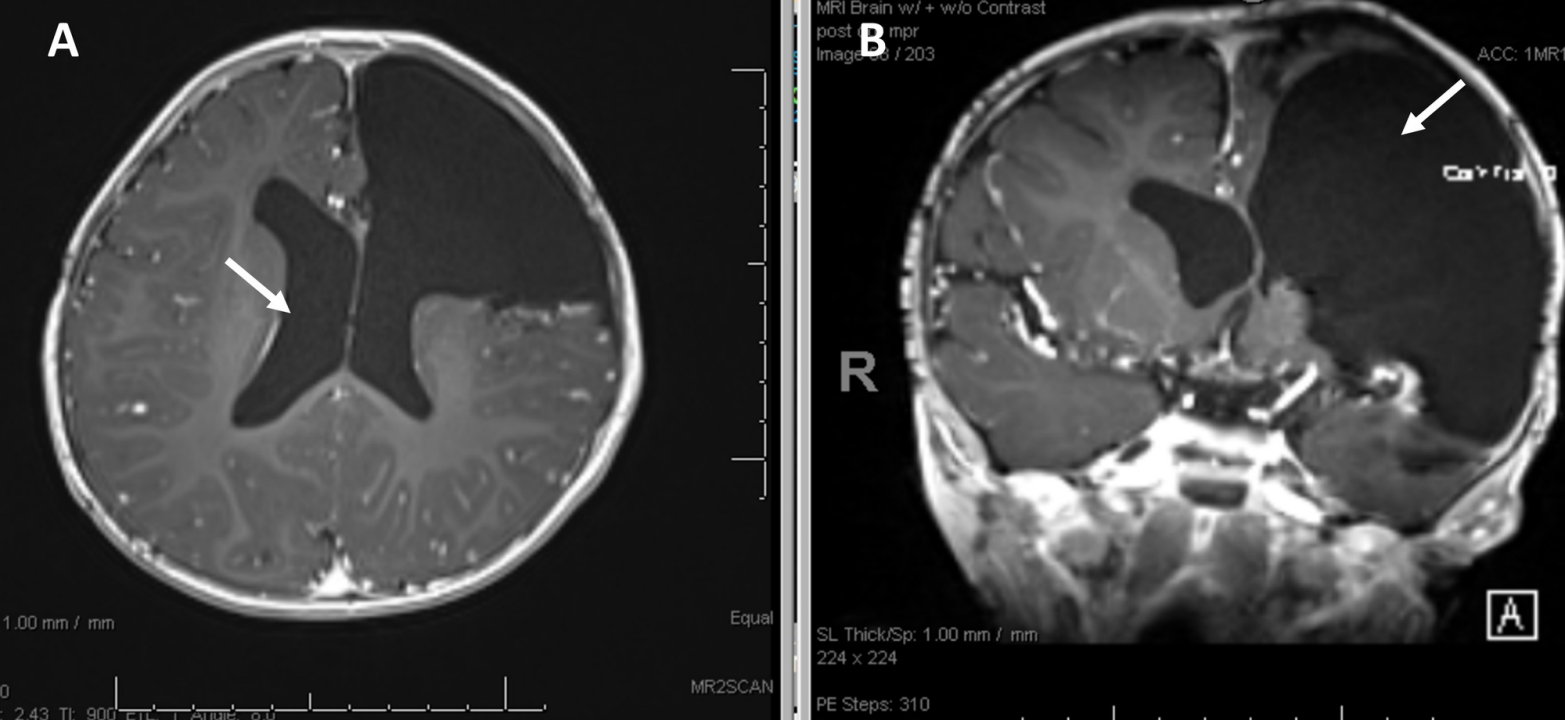
Imaging post second resection and continued chemotherapy MRIs status-post 15 months from the initial resection and 12 months post the second resection. (A) Axial MRI showing resolution of hydrocephalus (arrow). (B) Coronal MRI showing post-operative changes with no residual tumor (arrow)

She began three cycles of chemotherapy per Head Start III [10] (vincristine 0.05 mg/kg Days 1, 8, 15; cisplatin 3.5/mg/kg/day Day 1; cyclophosphamide 55 mg/kg Days 2&3; etoposide 4 mg/kg Days 2&3; methotrexate [MTX] 270 mg/kg - max 20 g) with leucovorin rescue. After these chemotherapy cycles, she underwent a second tumor resection. She then began high dose chemotherapy and received one dose of carboplatin 167mg/kg; however, concerns of wound infection prompted a return to the operating room for washout and further chemotherapy was held until her infection was fully treated. She resumed chemotherapy after she healed from her revision surgery. She also received an infusion of 2.8 X 106 CD 34+ cells/kg of previously harvested cryopreserved autologous bone marrow. She then began metronomic chemotherapy with cyclophosphamide, and etoposide. She remains stable and has been tolerating her treatment well. Her most recent imaging, 15 months after initial resection, continues to show no residual disease or tumor recurrence and no development of hydrocephalus or other sequelae (Figure 5A, B). 

## Discussion

The tumor described in this case study has an extremely unique pathology with features of different tumor types, including glial and ependymal. The diverse pathological features include L1CAM positive immunoreactivity, perivascular pseudo-rosettes, although few, and evident anaplasia with mitotic figures, pseudopallisading necrosis, and high MTB-1 labeling, suggestive of a high-grade neoplasm. Glial fibrillary acidic protein (GFAP) showed stout astrocyte processes grouped around vessels, typical for ependymoma. Supratentorial ependymomas have been found to frequently express the C11orf95-RELA fusion; however, this fusion was not found in our patient [11]. We do not routinely send for this mutation but due to the complexity of the histology, sequencing was performed to aid in finalizing a diagnosis. The EML4-ALK fusion and deletion of exons 14 to 19 of the ALK gene was noted with gene sequencing. This tumor has glial and ependymal features, and was classified as a high-grade neuroepithelial tumor with EML4-ALK fusion, reinforcing the impact of a molecular-based diagnosis in correct classification and management. The presence of the EML4-ALK mutation did not change the aggressiveness of the surgical approach as with a high-grade tumor like this; gross total resection is always desired.

In adults, EML4-ALK fusion tumors are more aggressive as these patients tend to present younger with more advanced disease. The EML4-ALK fusion is found more commonly in non-smoking patients who present with aggressive NSCLC [12,13]. Interestingly, in infantile high-grade glioma, ALK-fusion driven tumors may provide a favorable biology. Infantile high-grade glioma has a significantly better survival in comparison to older children with high-grade glioma [7,14]. Several of these large ALK-fusion driven infantile high-grade gliomas are congenital, and may have maturation/differentiation following chemotherapy [7,8,14]. Thus, in some cases, treatment with a less intense chemotherapeutic regimen may be warranted to limit overtly morbid therapy, especially with the availability of targeted agents; however, this should be individualized for the patient.

ALK tyrosine kinase inhibitors are continuously developed as the use of these inhibitors show improved disease control and tumor response rates compared to standard chemotherapy [15,16]. In this case, an ALK inhibitor was not used. There is currently no data that it would be effective in a patient with this mutation and it is not approved for infants. Logistically, it would also be nearly impossible to get an infant to swallow a capsule/pill, the only available formulation of ALK inhibitors. In addition to ALK-inhibitors, these tumors may respond to pan-TRK-inhibitors [17]. Further studies and clinical trials using these types of treatments are necessary to develop an effective therapeutic regimen for patients suffering from these types of tumors.

## Conclusions

This report describes a five-month-old female found to have an EML4-ALK fusion high-grade neuroepithelial tumor and her treatment course. This tumor has glial and ependymal features, and was classified as a high-grade neuroepithelial tumor with EML4-ALK fusion, reinforcing the impact of a molecular-based diagnosis in correct classification and management. This patient, underwent two tumor resections, is on a high dose chemotherapy regimen, and is currently doing well with no residual disease on MRI.
